# Protective potential of mesenchymal stem cells against COVID-19 during pregnancy

**DOI:** 10.2144/fsoa-2023-0179

**Published:** 2024-05-20

**Authors:** Sihem Aouabdi, Doaa Aboalola, Samer Zakari, Suliman Alwafi, Taoufik Nedjadi, Rawiah Alsiary

**Affiliations:** 1 King Abdullah International Medical Research Center, Jeddah, 21423, Saudi Arabia; 2 King Saud Bin Abdulaziz University for Health Sciences, Jeddah, 21423, Saudi Arabia

**Keywords:** COVID-19, MSCs, pregnancy, SARS-CoV-2, transplantation

## Abstract

SARS-CoV-2 causes COVID-19. COVID-19 has led to severe clinical illnesses and an unprecedented death toll. The virus induces immune inflammatory responses specifically cytokine storm in lungs. Several published reports indicated that pregnant females are less likely to develop severe symptoms compared with non-pregnant. Putative protective role of maternal blood circulating fetal mesenchymal stem cells (MSCs) has emerged and have been put forward as an explanation to alleviated symptoms. MSCs with immune-modulatory, anti-inflammatory and anti-viral roles, hold great potential for the treatment of COVID-19. MSCs could be an alternative to treat infections resulting from the SARS-CoV-2 and potential future outbreaks. This review focuses on the MSCs putative protective roles against COVID-19 in pregnant females.

## Plain language summary

The COVID-19 outbreak is still posing a global health concern. Despite the herd immunity provided by vaccination programs, no efficient treatments are yet available especially against the severe forms of the disease. According to published reports, pregnant females are less likely to develop the severe form of the disease due to the protective effect of specialized cells named mesenchymal stem cells (MSCs). These cells are present in the placenta and amniotic fluid. They can migrate from these tissues to the mother's blood stream and are believed to confer protection to the pregnant females against severe form of COVID-19. Further investigations are on the way to uncover the potential role of MSC as an alternative therapy for COVID-19 and other diseases.

## Tweetable abstract

The virus SARS-CoV-2 results in COVID-19 infection. Several reports indicated that pregnant females are less likely to develop severe symptoms. Circulating fetal Mesenchymal stem cells in pregnant females might protect them.

## Abstract (plain language summary)

SARS-CoV-2 result in highly contagious condition, the COVID-19. Symptoms can vary from asymptomatic, mild to severe respiratory distress. Antiviral, antimalarial, and anti-inflammatory medications were tested in COVID-19 treatment studies, but none of them were found to be effective. The available therapies and vaccinations are insufficient to fully guard against COVID-19 infection and its associated complications.

Since the COVID-19 outbreak, stem cells, in particular mesenchymal cells (MSCs), have received attention as an alternative therapy and are now emerging as a promising therapeutic option against COVID-19. Currently, MSCs are being used in more than 100 clinical trials to treat COVID-19 (source: Clinicaltrials.gov). According to several reports, patients in the majority of clinical trials using MSCs for the treatment of COVID-19 recovered well, with less lung damage and better survival. MSCs are a feasible treatment option for COVID-19 patients, particularly for those who are in a critical condition.

Several studies imply that pregnant women are less likely to get the severe form of COVID-19, although preclinical and clinical settings are still needed to confirm this. The number of critical cases of COVID-19 infection was extremely low in pregnant women. SARS-CoV-2 mother-to-fetus transmission has not been documented in the majority of COVID-19-pregnancy cases.

The presence of fetal MSCs in the mother's blood raises the possibility that MSCs from fetal tissues (placenta, amniotic fluid, cord blood) may shield the mothers from the potentially harmful effects of COVID-19 while they are pregnant. Maternal blood circulating fetal MSCs might be naturally safeguarding pregnant women.

MSCs, due to their anti-inflammatory, immunomodulatory, and antiviral activities, constitute a promising therapeutic target against SARS-CoV-2 induced inflammation. Lactoferrin may also defend against viral infection in females by lowering ACE2 together with the low expression of TMPRSS2 which are necessary for SARS-CoV-2 entry in the cells. The successful outcomes of MSCs clinical trials in COVID-19 patients, especially those in critical conditions, could be a treatment option for all including pregnant females, and those with underlying conditions. Clinical trials on the application of the fetal MSCs to pregnant females with COVID-19 would confirm the hypothesis.

## Graphical abstract

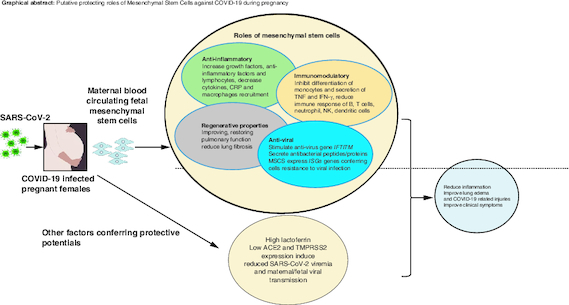

The first case of the COVID-19 was reported in late December 2019, in Wuhan, China [1,2]. The virus responsible for this disease was named by the International Committee on Taxonomy of Viruses as the SARS-CoV-2 [3]. COVID-19 is highly contagious and became a pandemic within few weeks from its first outbreak in China [4]. Incubation time of COVID-19 varies, ranging from 3–27 days [5]. The infection could last up to 6 weeks or longer if the patient has underlying health conditions [6].

The human to human fast spread of the COVID-19 makes it particularly concerning to health authorities. SARS-CoV-2 disseminated worldwide infecting around 237 million causing the death of more than 4 million [7]. There are currently few vaccines available and more are being developed targeting the new variants [8]. Vaccines are not enough to provide full protection against the COVID-19 infection as the current therapies showed no significant effects, especially on severe COVID-19 cases. Other therapies, which may hold great promises, need to be considered such as the application of the mesenchymal stem cells (MSCs).

Symptoms are varied, from asymptomatic or mild symptoms to pneumonia and severe respiratory failure. The most common symptoms of COVID-19 are high fever, tiredness, shortness of breath, dry cough, headache and sore throat. Other symptoms include a flu-like symptoms with runny nose, sneezing and diarrhea [1,2,9]. SARS-CoV-2 patients might also suffer from pneumonia and acute respiratory distress syndrome (ARDS), uncontrolled coagulation, metabolic acidosis, septic shock and multiple organ failure [10]. Patients with advanced age and with comorbidities are at higher risk of developing severe COVID-19 [11].

The cell entry mechanism of SARS-CoV-2 to the host cell surface is initiated by coupling the virus spike protein to the ACE2 receptors present on the epithelium of nose and the alveolar lung, thereby, leading to the respiratory symptoms [12,13]. In addition, the SARS-CoV-2 entry requires S protein priming via the TMPRSS2 that catalyzes the cleavage and fusion process [10]. The virus induces then inflammation, an immune response and cytokine storm in the lungs, which result in edema, disturbed oxygen exchange, hypoxia and ARDS, a life-threatening condition [1,14].

Currently, stem cells therapy is emerging as a potential therapeutic option against COVID-19. Stem cells especially MSCs, have been highlighted since the COVID-19 outbreak as an alternative therapy. For instance, these cells have previously shown to be effective in reducing pneumonia and pulmonary inflammation in mice infected with the avian influenza virus (H9N2) [14]. These cells are not only good candidate for allogeneic transplant, but they have immune-modulatory, anti-apoptotic and anti-inflammatory properties that point to their curative potential for treating inflammation and sepsis properties making them a potential therapy for treating COVID-19 [15,16].

It is documented that SARS-CoV-2 induces inflammation and decreases lymphocytes counts, therefore immunomodulation capabilities of MSCs in this respect to effectively inhibit the inflammation and increase the number of lymphocytes is being employed as an effective treatment [17]. There are a number of ongoing clinical trials exploring the use of MSCs for the treatment of coronavirus infection. It has been demonstrated that pregnant females with COVID-19 are less symptomatic and their intensive care unit (ICU) admission and respiratory distress rates are lower compared with non-pregnant females with COVID-19 [18].

Treatment trials for COVID-19 included antiviral, antimalarial and anti-inflammatory drugs, with no proven efficacy [19]. In the most critical cases, therapies recommended, including oxygen supply and mechanical ventilation, do not allow full recovery of the damaged lung function. In more than 70% of the cases at least one symptom will persist for several months after the infection [7]. Besides, the emergence of new variants that are not necessarily sensitive to the vaccines, more effective treatments need to be developed. Therefore, the potential of using stem cells as therapeutic target for critically ill COVID-19 patients is promising [20].

## Mesenchymal stem cells therapy for COVID-19

In 1968, Friedenstein and Phinney first isolated adult MSCs from bone marrow [21,22]. MSCs can also be found in other tissues such as adipose tissue, dental pulp and fetal tissues like placenta, umbilical cord, cord blood and amniotic fluid [23]. MSCs from adipose tissues, cord blood, placenta are easily isolated and are readily available. *In vitro*, MSCs are plastic adherent, express specific cell surface markers (CD105, CD73, CD90) and are multipotent [24]. They can be differentiated into adipocytes, chondrocytes and osteocytes depending on the signals received from the cell environment [25]. Beside their differentiation to these specific cell lines, MSCs can also differentiate into most cell types. MSCs do not express the MHCII which makes them nonimmunogenic, ideal for allogeneic transplant [26]. The regenerative properties of MSCs on injured tissues is either direct by cell-to-cell contacts or via paracrine signaling pathways [14]. Fetal MSCs are found in maternal blood as early as 7 weeks of pregnancy [27]. Due to their multipotentiality capacity, MSCs possess a great therapeutic potential in regenerative medicine for various diseases.

MSCs have shown promising results for the treatment of various conditions affecting the lungs, the heart like myocardial infarction, diabetes, hepatic failure, autoimmune diseases like multiple sclerosis and systemic lupus erythematous, and acute versus host disease [8,28]. Several clinical trials employing infusion of MSCs for the treatment of COVID-19 patients with lung inflammation and fibrosis showed promising results in terms of lung function recovery and patient survival with no adverse effects [8,29].

Previously, the allogeneic transplant of the MSCs reduced organ failure and ventilated patients affected by the ARDS [30]. MSCs have been used in clinical trials to treat patients with ARDS caused by the influenza virus H7N9. The transplant of the MSCs in these patients reduced significantly the death rate (17.6%) in the test group compared with control placebo group (54.5%) [31]. Currently, over 100 clinical trials are registered for the treatment of COVID-19 with MSCs (source: clinicaltrials.gov). It has been reported that most clinical trials using the MSCs for the treatment of the COVID-19 showed good recovery of the patients with decrease in lung damage and improved patient survival [7].

## Anti-inflammatory, immunomodulatory & antiviral roles of the MSCs

Because SARS-CoV-2 causes inflammation, MSCs could be a potential therapeutic target. It was previously shown that the injected MSCs reduced inflammation, pneumonia and lung injury resulting from the SARS infection [32,33]. There anti-inflammatory actions are mediated by modulating Treg cells and macrophages as well as alleviating cytokines proliferation [32]. They attenuate the recruitment of macrophages and mononuclear cells to the lungs together with increased IL10 levels [18]. The MSCs stimulate the transition of the inflammatory macrophage phenotype M1 to the anti-inflammatory and wound-healing M2 phenotype [34]. They trigger an increase in lymphocytes and a decrease in CRP correcting the decrease in lymphocytes and the increase in CRP caused by the COVID-19 [35]. Mesenchymal stem cells stimulate the secretion of growth factors like the Ang1, FGF-7, FGF-2, VEGF and HGF [8]. These factors are important in restoring the inflammatory and fibrosis damage caused by the COVID-19 in the lungs. Furthermore, MSCs inhibit the differentiation of monocytes into dendritic cells [36] and the secretion of TNF-α and IFN-γ by CD4^+^ T and CD8^+^ T cells [37]. The MSCs reduce the immune response of the B cells, T cells, neutrophils, natural killer (NK) cells and dendritic cells (DCs) [32]. Their immune-modulatory actions are also mediated by the TGFβ-1, HGF and INF-γ [38].

MSCs have antiviral actions mediated by the stimulation of antivirus gene *IFTITM* protecting the cells from viral invasion [39]. They secrete antibacterial peptide IL-37, human defensing-2 hepcidin and lipocalin-2 AMPs. They inhibit the synthesis of essential proteins, DNA and RNA of infected cells and regulate the infection and inflammation in COVID-19 patients [10]. MSCs express the *ISGs*, a factor that could induce resistance of MSCs to viral infection [17], so when injected, the cells will not be targeted by the SARS-CoV-2. Previous *in vivo* studies showed that the MSCs decreased the viral load in H1N1-infected animals [40].

MSCs represent a good therapeutic target against SARS-CoV-2-induced inflammation due to their anti-inflammatory, immunomodulatory and antiviral actions. Many clinical trials in phase I & II are ongoing for the application of the MSCs for the treatment of COVID-19 [14,41].

## COVID-19 infection in pregnancy

During pregnancy, there is a transient down regulation of the immune system modulated by the suppression of the T cell activity to maintain the tolerance to the pregnancy considered as an allograft [42]. Therefore, pregnant women are more vulnerable to viral infections as it was previously reported during the H1N1 influenza pandemic, the severe acute respiratory distress syndrome (SARS) and the Middle East respiratory distress syndrome (MERS) [42]. The exposure and severity of COVID-19 during pregnancy may be impacted by physiological, mechanical and immunological changes throughout pregnancy. To date, the data are insufficient to evaluate whether pregnancy enhances the susceptibility to SARS-CoV-2 infection; due to the lack of comparable incidence data and the difficulties in separating differences in susceptibility from various exposure risks. The limited number of clinical studies and living systematic reviews published on the potential risk factor of pregnancy and the severity of COVID-19 have been inconclusive. Few data support pregnancy as a risk factor for COVID-19-related severe disease; some of the best evidence comes from the US Centers for Disease Control and Prevention's COVID-19 surveillance system. The latter reported that pregnant women have higher risk of dying, being admitted to an intensive care unit, needing invasive ventilation and needing extracorporeal membrane oxygenation than non-pregnant women [43]. Thus far, the prevalence of neonatal COVID-19 positivity did not exceed the 5% [18]. It is interesting to note that in the majority of COVID-19-pregnancy instances, SARS-CoV-2 mother-to-fetus transmission has not been identified which might be due to the presence of lactoferrin in the placenta, amniotic fluid and lacteal secretions. Lactoferrin takes antiviral protection action by inducing host defense during pregnancy and downregulating the expression of the ACE2; therefor, there may not be much vertical transfer [44]. The intrauterine transmission is rare probably also due to the low SARS-CoV-2 viremia levels and the decline in the co-expression of the TMPRSS2 together with the ACE2 proteins which are necessary for SARS-CoV-2 entry into placental cells [43].

Nevertheless, during the outbreak of the COVID-19, pregnant females seemed to have less adverse events compared with the previous outbreak of the H1N1 and MERS [42]. The number of critical cases reported of pregnant women infected with SARS-CoV-2 was very low together with the number of infected newborns [18]. In a recent systematic meta-analysis published paper on COVID-19 pneumonia and SARS-CoV-2 positive pregnant women that included 87 SARS-CoV-2 positive pregnant women; 78% suffered from mild to moderate COVID-19 and 99.9% had successful deliveries. The overall proportions of vertical transmission, still birth and neonatal death were zero, 0.002 and 0.002, respectively; proposing a similar pattern of the clinical characteristics of COVID-19 pneumonia to that of other adult populations [45]. The severity of COVID-19 in pregnant women in terms of ICU upon admission, was similar to non-pregnant COVID-19 patients [46,47]. The presence of comorbidities in pregnant females with COVID-19 represented risk factor to develop moderate to severe infection [48]. The clinical features of the COVID-19 in pregnant women were similar to those observed in non-pregnant women [49]. In a study carried out in Wuhan, the percentage of pregnant women with severe COVID-19 (8%) was much lower than the pregnant women with mild symptoms (92%) [50]. Another study showed that the percentage of asymptomatic pregnant women was much higher (63.7%) than the symptomatic pregnant patients (36.3%) [51]. However, pregnant women who had symptoms (like fever and cough) were more likely to develop severe disease [52]. Centers for Disease Control and Prevention (CDC) reported higher death rates among symptomatic pregnant women [53]. The preterm birth risk was higher in symptomatic COVID-19 women compared with asymptomatic COVID-19 and uninfected pregnant females [51], although, the preterm birth was attributed to iatrogenic effects rather than resulting from COVID-19 infection. Wastnedge *et al.*, summarized data from 31 studies, involving 12,260 pregnant women with confirmed COVID-19 infection, on the outcomes of SARS-CoV-2 on pregnancy. The study showed the majority of females had mild to moderate symptoms and only minority were in critical care unit. Overall, there were 146 deaths in pregnant females, representing only 1.2% of all pregnant women infected with COVID-19 [54,55]. Another study carried out in Sweden showed that 65% of women in labor diagnosed positive for COVID-19 were asymptomatic [56]. Similar data were reported in a different study, where 61.4% of pregnant women were asymptomatic, 5% had severe COVID-19 and 97.5% of the newborns were negative for SARS-CoV-2 immediately after birth [57]. Dashraath *et al.*, study showed no such deaths in pregnant women diagnosed with COVID-19 when compared with pregnant women with SARS and MERS [58]. The percentage of mechanical ventilation was 2% in pregnant women with COVID-19 compared with 35% and 41% in women with SARS and MERS, respectively [58]. Another study revealed no deaths of pregnant COVID-19 patients [59]. This study showed that 76.7% of respiratory injury in pregnant women with shorter time-to-discharge compared with 92.5% of respiratory injury in non-pregnant women. However, a meta-analysis study showed that pregnant women are more likely to be admitted in ICU compared with non-pregnant women [60]. The discrepancies in these data is probably due to the study protocol that is different from country to country in terms of care for pregnant women with COVID-19. Table 1 shows data from various studies carried out in different countries on the outcome of COVID-19 on pregnant females. The study population (country), the number of pregnant females testing positive for SARS-CoV-2, their age, age of pregnancy, patient's symptoms (absence or presence of symptoms and their intensity), the outcomes on pregnancy, the ICU status, delivery status, and neonatal SARS-CoV-2 status are summarized. The studies showed that the ICU admission was from no cases up to 10% of the cases admitted in ICU. Females suffered from preterm delivery with more recorded caesarean during the pandemic [51,52]. Transmission from mother to newly born babies was also very low.

**Table 1.** Summary report highlighting the clinical outcomes of COVID-19 in pregnant females.

Study population (country)	Cases SARS-CoV-2 positive (N)	Age (years)	Age of pregnancy	Asymptomatic/mild/moderate	Symptomatic/severe symptoms	Outcomes on pregnancy	ICU admission	Delivery status	Neonatal SARS-CoV-2	Ref.
USA	4,145	27–35	<24 and >24 weeks	Recorded only critical cases	32	No maternal/fetal deaths, high preterm delivery, high cesarean delivery	32 cases	19 deliveries, 18 (94.7%) preterm, 11 (57.9%) delivered in ICU,	Not reported	[46]
USA	8207	15–44	Not reported	Asymptomatic (2.9%) similar to non-pregnant females	97.1% symptomatic similar to non-pregnant females (fever, cough, diarrhea, runny nose)	31.5% hospitalized 0.2% death, similar to non-pregnant females (0.2), no increase in mortality rate for pregnant females	1.5% pregnant vs 0.9% in non-pregnant females) with underlying conditions (diabetes, cardiovascular disease)	Not reported	Not reported	[47]
Japan	254	26–35	13–31 weeks	224 patients (88%) mild symptoms	30 patients (12%) severe symptoms	Moderate to severe infection in presence of comorbidities (diabetes, obesity, smoking, pre-eclampsia)	No admissions	Not reported	Not reported	[48]
Cross countries	295	20–44	5–41 weeks	Third of patients asymptomatic or mild symptoms	0–14% sever pneumonia	3% (9) deaths 1 neonatal death	4.7% cases	219 deliveries (78.1% cesarean)	8.7% (19 cases out of 219) positive SARS-CoV-2	[49]
China	118	28–34	75 (64%) in third trimester	Mild symptoms (fever, cough) 109 (92%)	Severe symptoms (hypoxemia) 9 (8%)	No deaths, 94% discharged	1 of 9 with severe symptoms	68 (58%) gave birth, 3 abortions, 2 ectopic pregnancies, 4 induced abortions	No positive SARS-CoV-2	[50]
UK	214	26–34	First trimester to delivery	Asymptomatic 63.7%	Symptomatic 36.3%, pre-eclampsia 2.8%	More caesarean recorded during pandemic	4 (1.9%)	Preterm birth in symptomatic patients, no neonatal death related to COVID-19	11% positive for SARS-CoV-2	[51]
Cross countries	26528	18–44	First to third trimester	7.5–32.6% asymptomatic	Symptoms (rates differ for symptoms, from 6% for myalgia to 87.5% for fever	1.4–12% likely to develop severe disease, maternal mortality <2% – neonatal mortality <3% – stillbirth <2.5%, neonatal ICU 3.1–76.9%	3–10% admissions. 1.4–5.5% mechanical ventilation rates neonatal ICU admission 3.1–76.9%	Caesarean (term and pre-term) 52.3–95.8%,	1.6–10% positive SARS-CoV-2	[52]
Sweden	155	13–>35	In labor	65% asymptomatic	35% symptomatic presented pre-eclampsia	Pre-eclampsia, gestational diabetes, preterm birth <37 weeks, post-partum hemorrhage, emergency cesarean 1 stillbirth,	No admissions	Spontaneous (69%), instrumental (9%), cesarean (planned 13.5%, emergency 9.7%)	Not reported	[56]
USA	241	18–47	Third trimester	61.4% asymptomatic	26.1% severe, 5% critical symptoms	Cesarean for symptomatic, mild, severe and critical cases (33.3%, 34.4% 52.4%, 91.7%, respectively) no maternal death	7.1%, admissions 3.7% intubation during delivery	99.2% successful delivery-	2.5% positive SARS-CoV-2	[57]
Singapore	55	23–40	Third trimester except for 2 (<28 weeks)	Mostly mild symptoms, fever, cough, dyspnea	2% severe COVID-19	No mortality, miscarriage (2%), preterm birth (43%) neonatal death (2%)	Mechanical ventilation (2%),	Miscarriage, preterm birth	4.4% (2 cases out of 46 neonates) positive SARS-CoV-2	[58]
China	30	30–34	Not reported	76.6% respiratory system injury (fever, cough, sputum)	Abnormal heart, liver digestive function (3.3–20%),	No deaths, 76.7% low respiratory system injury vs 92.5% in non-pregnant females terminated pregnancies 22 (73.3%), 8 (26.7%) continued	No admissions	Not reported	Not reported	[59]

ICU: Intensive care unit; N: Number; Ref.: Reference.

Altogether, the current data suggest that pregnant women are less vulnerable to develop the severe form of the COVID-19, though, this needs to be validated in preclinical and clinical setting.

## Clinical applications of MSCs for COVID-19 & their potential protection in SARS-CoV-2-infected pregnant females

**Figure 1.**
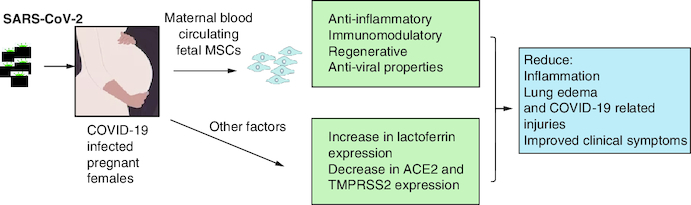
Factors putatively protecting COVID-19 pregnant females. Maternal blood circulating fetal mesenchymal stem cells (MSCS), lactoferrin lead to reducing COVID-19-related symptoms.

MSCs have been used for decades in clinical trials for the treatment of many conditions including respiratory infections like for instance acute respiratory distress syndrome (ARDS) with promising results and with no major side effects [61,62]. Therefore, the use of MSCs in critically ill COVID-19 patients is very promising. Many countries have registered and initiated their clinical trials for the treatment with MSCs for COVID-19 infection and its complications (source: clinicaltrials.gov). The results demonstrated that MSC therapy is effective in alleviating immune response due to SARS-CoV-2, reducing lung inflammation and stimulating their regeneration [14,35,63]. Published reports revealed that injected MSCs homed in on the site of the mostly infected organ released anti-inflammatory factors by modulating regulatory T cells and macrophages and alleviated the proliferation of cytokines [32]. Shanchez-Guijo *et al.* demonstrated that MSCs injection to 13 mechanically ventilated patients secondary to COVID-19 pneumonia significantly improved patients' outcome, where almost half of the patients were extubated at day 16 [64]. Furthermore, patients treated with MSCs showed improved lung functions, x-rays findings and reduced inflammatory markers [65]. Large cohort and long term studies are needed to ensure safety and efficacy of the treatment with MSCs [63,66]. As discussed earlier, it is suggested that pregnant females are less likely to have severe form of the COVID-19 infection. The presence of fetal MSCs in the mother's blood suggest that the MSCs from fetal tissues (placenta, amniotic fluid, cord blood) might protect the mothers against the serious outcomes of the COVID-19 during pregnancy [18]. Lactoferrin might also protect females from viral infection by reducing the expression of the ACE2, together with the down expression of TMPRSS2 as stated earlier. Figure 1 shows the putative protecting roles of the maternal blood circulating fetal MSCs and other factors such as lactoferrin, ACE2 and TMPRSS2.

Few approaches might be applied to test the hypothesis of the protective potentials of the fetal MSCs in COVID-19 pregnant females. Analysis of the proteomic, transcriptomic, genetic and epigenetic of the blood from pregnant females with COVID-19 to determine specific genes, epigenetic components, proteins, or processes involved in fetal MSC presence and functions. Examine the modulatory effects of fetal MSCs on maternal immunological responses in pregnant females with COVID-19 in terms of cytokines and anti-inflammatory markers. Evaluate the effects of injected fetal MSCs in *in vivo* model of COVID-19 pregnant animals and comparing them to healthy pregnant animals and their counterpart of non-pregnant. The injected MSCs could be tagged to follow their homing capacity using an *in vivo* animal imaging to test their roles in various tissues and organs.

Gentile *et al.* suggested two protocols for the treatment of the COVID-19 patients, the first is the emergency protocol and the second is the consolidated administration [10]. In the first protocol, cells can be acquired from an approved authority for example the Food and Drug Administration (FDA), GMP laboratory, or European approved labs or tissue bank. In the second protocol, the cell infusion could be autologous or allogeneic.

## Conclusion

MSCs are a promising tool for the treatment of COVID-19 patients especially those in critical status. The presence of fetal MSCs in maternal blood are believed to reduce the severeness of COVID-19 related symptoms in pregnant women indicating that MSCs might be naturally protective. The positive outcomes of the MSCs transplantation to severely ill patients affected by the COVID-19, might be the solution for the treatment of COVID-19 patients. Large randomized multicenter clinical trials are necessary to validate the recent findings and to set the ground for solid application of MSCs in clinical practice.

## Future perspective

In the last decades the application of MSCs in clinical trials for various conditions have advanced greatly owing to their immunomodulatory characteristics [62]. During the COVID-19 outbreak, the application of MSCs as a treatment, especially in critically ill patients, showed promising results with no major adverse effects. Given the contagious nature of the SARS-CoV-2 and the possibility of future outbreaks from other viruses, MSCs represent a great potential for the treatment of COVID-19, especially that no definite treatment was successful in treating critical cases of the infection. The role of the MSCs in protecting the pregnant females need to be investigated in more depth. These studies could be designed as either an autologous or an allogeneic transplantation of the MSCs. To effectively utilize MSCs in the fight against COVID-19, cooperation between researchers, clinicians, and health organizations is essential. Standardized protocols have to be implemented to comprehensively conclude the clinical efficacy and safety of MSCs. The conditions could be optimized to test the protective potentials of the MSCs and their use in clinical trials in pregnant females with COVID-19. In the 10 years, MSCs applications will benefit the pregnant females and the general population, especially those categories suffering from underlying comorbidities where no medical treatment nor vaccination can help.

## References

[1] Huang C, Wang Y, Li X et al. Clinical features of patients infected with 2019 novel coronavirus in Wuhan, China. Lancet 395(10223), 497–506 (2020). 31986264 10.1016/S0140-6736(20)30183-5 PMC7159299

[2] Nadeem MS, Zamzami MA, Choudhry H et al. Origin, potential therapeutic targets and treatment for coronavirus disease (COVID-19). Pathogens 9(4), 307 (2020). 32331255 10.3390/pathogens9040307 PMC7238035

[3] Cui J, Li F, Shi ZL. Origin and evolution of pathogenic coronaviruses. Nat Rev Microbiol 17(3), 181–192 (2019). 30531947 10.1038/s41579-018-0118-9 PMC7097006

[4] Guan W-J, Ni Z-Y, Hu Y et al. Clinical characteristics of 2019 novel coronavirus infection in China. medRxiv doi: 10.1101/2020.02.06.20020974 (2020) (Epub ahead of print).

[5] Lauer SA, Grantz KH, Bi Q et al. The incubation period of coronavirus disease 2019 (COVID-19) from publicly reported confirmed cases: estimation and application. Ann. Intern. Med. 172(9), 577–582 (2020). 32150748 10.7326/M20-0504 PMC7081172

[6] Munster VJ, Koopmans M, van Doremalen N, van Riel D, de Wit E. A novel coronavirus emerging in china - key questions for impact assessment. N. Engl. J. Med. 382(8), 692–694 (2020). 31978293 10.1056/NEJMp2000929

[7] Shi L, Yuan X, Yao W et al. Human mesenchymal stem cells treatment for severe COVID-19: 1-year follow-up results of a randomized, double-blind, placebo-controlled trial. EBioMedicine 75, 103789 (2022). 34963099 10.1016/j.ebiom.2021.103789 PMC8709782

[8] Karakas N, Ucuncuoglu S, Uludag D, Karaoglan BS, Shah K, Ozturk G. Mesenchymal stem cell-based COVID-19 therapy: bioengineering perspectives. Cells 11(3), 465 (2022). 35159275 10.3390/cells11030465 PMC8834073

[9] Wang C, Horby PW, Hayden FG, Gao GF. A novel coronavirus outbreak of global health concern. Lancet 395(10223), 470–473 (2020). 31986257 10.1016/S0140-6736(20)30185-9 PMC7135038

[10] Gentile P, Sterodimas A, Pizzicannella J, Calabrese C, Garcovich S. Research progress on mesenchymal stem cells (MSCs), adipose-derived mesenchymal stem cells (AD-MSCs), drugs, and vaccines in inhibiting COVID-19 disease. Aging Dis 11(5), 1191–1201 (2020). 33014532 10.14336/AD.2020.0711 PMC7505274

[11] Wu Z, McGoogan JM. Characteristics of an important lessons from the coronavirus disease 2019 (COVID-19) outbreak in china: summary of a report of 72–314 cases from the chinese center for disease control and prevention. JAMA 323(13), 1239–1242 (2020). 32091533 10.1001/jama.2020.2648

[12] Shereen MA, Khan S, Kazmi A, Bashir N, Siddique R. COVID-19 infection: emergence, transmission, and characteristics of human coronaviruses. Journal of Advanced Research 24, 91–98 (2020). 32257431 10.1016/j.jare.2020.03.005 PMC7113610

[13] Zhao Y, Zhao Z, Wang Y, Zhou Y, Ma Y, Zuo W. Single-Cell RNA expression profiling of ACE2, the receptor of SARS-CoV-2. Am. J. Respir. Crit. Care Med. 202(5), 756–759 (2020). 32663409 10.1164/rccm.202001-0179LE PMC7462411

[14] Alzahrani FA, Saadeldin IM, Ahmad A et al. The potential use of mesenchymal stem cells and their derived exosomes as immunomodulatory agents for COVID-19 patients. Stem cells international 2020, 8835986 (2020). 33014070 10.1155/2020/8835986 PMC7512102

[15] Zheng G, Huang R, Qiu G et al. Mesenchymal stromal cell-derived extracellular vesicles: regenerative and immunomodulatory effects and potential applications in sepsis. Cell Tissue Res. 374(1), 1–15 (2018). 29955951 10.1007/s00441-018-2871-5

[16] Su N, Gao PL, Wang K, Wang JY, Zhong Y, Luo Y. Fibrous scaffolds potentiate the paracrine function of mesenchymal stem cells: a new dimension in cell-material interaction. Biomaterials 141, 74–85 (2017). 28667901 10.1016/j.biomaterials.2017.06.028

[17] Majolo F, da Silva GL, Vieira L, Timmers L, Laufer S, Goettert MI. Review of trials currently testing stem cells for treatment of respiratory diseases: facts known to date and possible applications to COVID-19. Stem Cell Rev Rep 17(1), 44–55 (2021). 32827081 10.1007/s12015-020-10033-6 PMC7442550

[18] Samara A, Herlenius E. Is there an effect of fetal mesenchymal stem cells in the mother-fetus dyad in COVID-19 pregnancies and vertical transmission? Front Physiol 11, 624625 (2020). 33679426 10.3389/fphys.2020.624625 PMC7928412

[19] Hadi A, Kadhom M, Hairunisa N, Yousif E, Mohammed S. A review on COVID-19: origin, spread, symptoms, treatment and prevention. Biointerface Research in Applied Chemistry 10, 7234–7242 (2020).

[20] Chrzanowski W, Kim SY, McClements L. Can stem cells beat COVID-19: advancing stem cells and extracellular vesicles toward mainstream medicine for lung injuries associated with SARS-CoV-2 infections. Front Bioeng Biotechnol 8, 554 (2020). 32574317 10.3389/fbioe.2020.00554 PMC7264098

[21] Friedenstein AJ, Petrakova KV, Kurolesova AI, Frolova GP. Heterotopic of bone marrow. Analysis of precursor cells for osteogenic and hematopoietic tissues. Transplantation 6(2), 230–247 (1968). 5654088

[22] Phinney DG. Building a consensus regarding the nature and origin of mesenchymal stem cells. Journal of cellular biochemistry. Supplement 38, 7–12 (2002). 12046852 10.1002/jcb.10084

[23] Main H, Munsie M, O'Connor MD. Managing the potential and pitfalls during clinical translation of emerging stem cell therapies. Clin Transl Med 3, 10 (2014). 24949190 10.1186/2001-1326-3-10 PMC4049443

[24] Dominici M, Le Blanc K, Mueller I et al. Minimal criteria for defining multipotent mesenchymal stromal cells. The International Society for Cellular Therapy position statement. Cytotherapy 8(4), 315–317 (2006). 16923606 10.1080/14653240600855905

[25] Pittenger MF, Mackay AM, Beck SC et al. Multilineage potential of adult human mesenchymal stem cells. Science 284(5411), 143–147 (1999). 10102814 10.1126/science.284.5411.143

[26] Li C, Zhang W, Jiang X, Mao N. Human-placenta-derived mesenchymal stem cells inhibit proliferation and function of allogeneic immune cells. Cell Tissue Res. 330(3), 437–446 (2007). 17899199 10.1007/s00441-007-0504-5

[27] O'Donoghue K, Choolani M, Chan J et al. Identification of fetal mesenchymal stem cells in maternal blood: implications for non-invasive prenatal diagnosis. Mol Hum Reprod 9(8), 497–502 (2003). 12837927 10.1093/molehr/gag063

[28] Li TS, Hayashi M, Ito H et al. Regeneration of infarcted myocardium by intramyocardial implantation of *ex vivo* transforming growth factor-beta-preprogrammed bone marrow stem cells. Circulation 111(19), 2438–2445 (2005). 15883211 10.1161/01.CIR.0000167553.49133.81

[29] Tang L, Jiang Y, Zhu M et al. Clinical study using mesenchymal stem cells for the treatment of patients with severe COVID-19. Front Med 14(5), 664–673 (2020). 32761491 10.1007/s11684-020-0810-9 PMC7406954

[30] Matthay MA, Calfee CS, Zhuo H et al. Treatment with allogeneic mesenchymal stromal cells for moderate to severe acute respiratory distress syndrome (START study): a randomised phase IIa safety trial. Lancet Respir Med 7(2), 154–162 (2019). 30455077 10.1016/S2213-2600(18)30418-1 PMC7597675

[31] Chen J, Hu C, Chen L et al. Clinical study of mesenchymal stem cell treatment for acute respiratory distress syndrome induced by epidemic influenza A (H7N9) infection: a hint for COVID-19 treatment. Engineering (Beijing) 6(10), 1153–1161 (2020). 32292627 10.1016/j.eng.2020.02.006 PMC7102606

[32] Du YM, Zhuansun YX, Chen R, Lin L, Lin Y, Li JG. Mesenchymal stem cell exosomes promote immunosuppression of regulatory T cells in asthma. Exp. Cell Res. 363(1), 114–120 (2018). 29277503 10.1016/j.yexcr.2017.12.021

[33] Li F. Structure, Function, and Evolution of Coronavirus Spike Proteins. Annu Rev Virol 3(1), 237–261 (2016). 27578435 10.1146/annurev-virology-110615-042301 PMC5457962

[34] Manning CN, Martel C, Sakiyama-Elbert SE et al. Adipose-derived mesenchymal stromal cells modulate tendon fibroblast responses to macrophage-induced inflammation *in vitro*. Stem Cell Res Ther 6(1), 74 (2015). 25889287 10.1186/s13287-015-0059-4 PMC4416344

[35] Leng Z, Zhu R, Hou W et al. Transplantation of ACE2(-) mesenchymal stem cells improves the outcome of patients with COVID-19 pneumonia. Aging Dis 11(2), 216–228 (2020). 32257537 10.14336/AD.2020.0228 PMC7069465

[36] Al Jumah MA, Abumaree MH. The immunomodulatory and neuroprotective effects of mesenchymal stem cells (MSCs) in experimental autoimmune encephalomyelitis (EAE): a model of multiple sclerosis (MS). Int J Mol Sci 13(7), 9298–9331 (2012). 22942767 10.3390/ijms13079298 PMC3430298

[37] Zheng ZH, Li XY, Ding J, Jia JF, Zhu P. Allogeneic mesenchymal stem cell and mesenchymal stem cell-differentiated chondrocyte suppress the responses of type II collagen-reactive T cells in rheumatoid arthritis. Rheumatology (Oxford) 47(1), 22–30 (2008). 18077486 10.1093/rheumatology/kem284

[38] Gentile P, Sterodimas A. Adipose-derived stromal stem cells (ASCs) as a new regenerative immediate therapy combating coronavirus (COVID-19)-induced pneumonia. Expert Opin Biol Ther 20(7), 711–716 (2020). 32329380 10.1080/14712598.2020.1761322 PMC7196919

[39] Xu Z, Shi L, Wang Y et al. Pathological findings of COVID-19 associated with acute respiratory distress syndrome. Lancet Respir Med 8(4), 420–422 (2020). 32085846 10.1016/S2213-2600(20)30076-X PMC7164771

[40] Khatri M, Richardson LA, Meulia T. Mesenchymal stem cell-derived extracellular vesicles attenuate influenza virus-induced acute lung injury in a pig model. Stem Cell Res Ther 9(1), 17 (2018). 29378639 10.1186/s13287-018-0774-8 PMC5789598

[41] Abu-El-Rub E, Khasawneh RR, Almahasneh F et al. Mesenchymal stem cells and COVID-19: what they do and what they can do. World journal of stem cells 13(9), 1318–1337 (2021). 34630865 10.4252/wjsc.v13.i9.1318 PMC8474724

[42] Qiancheng X, Jian S, Lingling P et al. Coronavirus disease 2019 in pregnancy. Int J Infect Dis 95, 376–383 (2020). 32353549 10.1016/j.ijid.2020.04.065 PMC7185021

[43] Jamieson DJ, Rasmussen SA. An update on COVID-19 and pregnancy. Am. J. Obstet. Gynecol. 226(2), 177–186 (2022). 34534497 10.1016/j.ajog.2021.08.054 PMC8438995

[44] Naidu SAG, Clemens RA, Pressman P, Zaigham M, Davies KJA, Naidu AS. COVID-19 during pregnancy and postpartum. J. Diet Suppl. 19(1), 78–114 (2022). 33164606 10.1080/19390211.2020.1834047

[45] Kasraeian M, Zare M, Vafaei H et al. COVID-19 pneumonia and pregnancy; a systematic review and meta-analysis. J Matern Fetal Neonatal Med 35(9), 1652–1659 (2022). 32429786 10.1080/14767058.2020.1763952

[46] Easter SR, Gupta S, Brenner SK, Leaf DE. Outcomes of critically Ill pregnant women with COVID-19 in the United States. Am. J. Respir. Crit. Care Med. 203(1), 122–125 (2021). 33026829 10.1164/rccm.202006-2182LE PMC7781146

[47] Ellington S, Strid P, Tong VT et al. Characteristics of women of reproductive age with laboratory-confirmed SARS-CoV-2 infection by pregnancy status - United States, January 22–June 7, 2020. MMWR Morb. Mortal. Wkly Rep. 69(25), 769–775 (2020). 32584795 10.15585/mmwr.mm6925a1 PMC7316319

[48] Shoji K, Tsuzuki S, Akiyama T et al. Clinical characteristics and outcomes of coronavirus disease 2019 (COVID-19) in pregnant women: a propensity score-matched analysis of data from the COVID-19 Registry Japan. Clin. Infect. Dis. 75(1), e397–e402 (2022). 35037051 10.1093/cid/ciac028 PMC8807242

[49] Juan J, Gil MM, Rong Z, Zhang Y, Yang H, Poon LC. Effect of coronavirus disease 2019 (COVID-19) on maternal, perinatal and neonatal outcome: systematic review. Ultrasound Obstet. Gynecol. 56(1), 15–27 (2020). 32430957 10.1002/uog.22088 PMC7276742

[50] Chen L, Li Q, Zheng D et al. Clinical characteristics of pregnant women with Covid-19 in Wuhan, China. N. Engl. J. Med. 382(25), e100 (2020). 32302077 10.1056/NEJMc2009226 PMC7182016

[51] Wilkinson M, Johnstone ED, Simcox LE, Myers JE. The impact of COVID-19 on pregnancy outcomes in a diverse cohort in England. Sci Rep 12(1), 942 (2022). 35042979 10.1038/s41598-022-04898-5 PMC8766432

[52] Papapanou M, Papaioannou M, Petta A et al. Maternal and neonatal characteristics and outcomes of COVID-19 in pregnancy: an overview of systematic reviews. Int J Environ Res Public Health 18(2), 596 (2021). 33445657 10.3390/ijerph18020596 PMC7828126

[53] Zambrano LD, Ellington S, Strid P et al. Update: characteristics of symptomatic women of reproductive age with laboratory-confirmed SARS-CoV-2 Infection by pregnancy status - United States, January 22–October 3, 2020. MMWR Morb. Mortal. Wkly Rep. 69(44), 1641–1647 (2020). 33151921 10.15585/mmwr.mm6944e3 PMC7643892

[54] Badell ML, Dude CM, Rasmussen SA, Jamieson DJ. Covid-19 vaccination in pregnancy. BMJ 378, e069741 (2022). 35948352 10.1136/bmj-2021-069741 PMC9363819

[55] Wastnedge EAN, Reynolds RM, van Boeckel SR et al. Pregnancy and COVID-19. Physiol. Rev. 101(1), 303–318 (2021). 32969772 10.1152/physrev.00024.2020 PMC7686875

[56] Ahlberg M, Neovius M, Saltvedt S et al. Association of SARS-CoV-2 test status and pregnancy outcomes. JAMA 324(17), 1782–1785 (2020). 32965467 10.1001/jama.2020.19124 PMC7512127

[57] Khoury R, Bernstein PS, Debolt C et al. Characteristics and outcomes of 241 births to women with severe acute respiratory syndrome coronavirus 2 (SARS-CoV-2) infection at five New York City medical centers. Obstet. Gynecol. 136(2), 273–282 (2020). 32555034 10.1097/AOG.0000000000004025

[58] Dashraath P, Wong JLJ, Lim MXK et al. Coronavirus disease 2019 (COVID-19) pandemic and pregnancy. Am. J. Obstet. Gynecol. 222(6), 521–531 (2020). 32217113 10.1016/j.ajog.2020.03.021 PMC7270569

[59] Zha Y, Chen G, Gong X et al. Coronavirus disease 2019 in pregnant and non-pregnant women: a retrospective study. Chin Med J (Engl) 134(10), 1218–1220 (2021). 34018998 10.1097/CM9.0000000000001396 PMC8143774

[60] Khan DSA, Pirzada AN, Ali A, Salam RA, Das JK, Lassi ZS. The differences in clinical presentation, management, and prognosis of laboratory-confirmed COVID-19 between pregnant and non-pregnant women: a systematic review and meta-analysis. Int J Environ Res Public Health 18(11), 5613 (2021). 34074005 10.3390/ijerph18115613 PMC8197383

[61] Zheng G, Huang L, Tong H et al. Treatment of acute respiratory distress syndrome with allogeneic adipose-derived mesenchymal stem cells: a randomized, placebo-controlled pilot study. Respir Res 15(1), 39 (2014). 24708472 10.1186/1465-9921-15-39 PMC3994204

[62] Zhou T, Yuan Z, Weng J et al. Challenges and advances in clinical applications of mesenchymal stromal cells. J Hematol Oncol 14(1), 24 (2021). 33579329 10.1186/s13045-021-01037-x PMC7880217

[63] Chen L, Qu J, Kalyani FS et al. Mesenchymal stem cell-based treatments for COVID-19: status and future perspectives for clinical applications. Cell. Mol. Life Sci. 79(3), 142 (2022). 35187617 10.1007/s00018-021-04096-y PMC8858603

[64] Sanchez-Guijo F, Garcia-Arranz M, Lopez-Parra M et al. Adipose-derived mesenchymal stromal cells for the treatment of patients with severe SARS-CoV-2 pneumonia requiring mechanical ventilation. A proof of concept study. EClinicalMedicine 25, 100454 (2020). 32838232 10.1016/j.eclinm.2020.100454 PMC7348610

[65] Qu W, Wang Z, Hare JM et al. Cell-based therapy to reduce mortality from COVID-19: systematic review and meta-analysis of human studies on acute respiratory distress syndrome. Stem Cells Transl Med 9(9), 1007–1022 (2020). 32472653 10.1002/sctm.20-0146 PMC7300743

[66] Chouw A, Milanda T, Sartika CR, Kirana MN, Halim D, Faried A. Potency of mesenchymal stem cell and its secretome in treating COVID-19. Regen Eng Transl Med 8(1), 43–54 (2022). 33723519 10.1007/s40883-021-00202-5 PMC7945610

